# Gastric fundus leiomyoma: a rare case report

**DOI:** 10.3389/fonc.2025.1652318

**Published:** 2025-08-15

**Authors:** Kaiyuan Luo, Jianfeng Bin, Jun Bian, Haomin Wang, Dai Tang, Maozhao Yan, Hua Ge

**Affiliations:** Department of Gastrointestinal Surgery, Third Affiliated Hospital of Zunyi Medical University (The First People’s Hospital of Zunyi), Zunyi, Guizhou, China

**Keywords:** gastric leiomyoma, gastrointestinal stromal tumors, case report, radiological diagnosis, laparoscopic surgery, cardia-sparing resection

## Abstract

**Background:**

Gastric leiomyoma is a rare, slow-growing benign tumor originating from the smooth muscle cells of the gastric wall. It is typically asymptomatic and presents insidiously. Due to its clinical and imaging features resembling those of gastrointestinal stromal tumors and other gastric neoplasms, it is often misdiagnosed, leading to unnecessary surgical interventions. This article reports a case of gastric leiomyoma in a 28-year-old male, aiming to explore its diagnosis, differential diagnosis, and treatment strategies, emphasizing the importance of comprehensive preoperative imaging evaluation to provide a reference for clinical practice.

**Case presentation:**

A 28-year-old male underwent gastroscopy due to abdominal pain, which revealed a submucosal protrusion in the gastric fundus. Imaging studies (computed tomography, endoscopic ultrasonography, and magnetic resonance imaging) showed a round-like mass approximately 6.3 cm in diameter in the gastric fundus, with mixed internal echogenicity. The patient underwent laparoscopic resection of the gastric fundus tumor, during which the tumor was completely excised. Postoperative pathological and immunohistochemical results confirmed the diagnosis of gastric leiomyoma. The patient recovered well and was discharged one week after surgery.

**Conclusions:**

The diagnosis of gastric leiomyoma requires a combination of imaging, pathological, and immunohistochemical findings to differentiate it from other gastric tumors such as gastrointestinal stromal tumors. Preoperative comprehensive analysis using computed tomography, magnetic resonance imaging, and endoscopic ultrasonography can improve diagnostic accuracy. For gastric leiomyoma, tumor resection with preservation of the cardia is an ideal surgical approach, ensuring negative margins while reducing postoperative complications (anastomotic stenosis). The successful treatment of this case provides a reference for the diagnosis and surgical management of gastric leiomyoma, contributing to improved patient prognosis and quality of life.

## 1 Introduction

Leiomyomas are classified as a subgroup of smooth muscle tumor and most commonly occur in the esophagus, colon, and rectum. When detected in the gastrointestinal tract, these tumors are typically benign, and complete surgical resection is associated with no risk of recurrence or metastasis (1). The observation of mitotic activity during histological examination should raise the consideration of a malignant tumor. Gastric leiomyoma is a benign tumor of mesenchymal origin that occurs in the stomach and is relatively rare in clinical practice. The majority of cases originate from the muscularis propria, whereas a minority arise from the muscularis mucosae. These tumors are most frequently located in the cardia and commonly involve the esophagogastric junction. The development of gastric leiomyoma is insidious, and with no obvious clinical symptoms during the early stages. The lesions are predominantly solitary but may occasionally be multiple. With gradual tumor enlargement, symptoms such as abdominal discomfort and incomplete obstruction of the cardia may develop (2). In the early stages of the disease, gastric leiomyoma lacks specific indicators, making it easily confused with conditions such as gastric ulcers, gastric polyps, and malignant gastric tumors. Gastric leiomyoma is a type of mesenchymal tumor of the stomach. Mesenchymal tumors of the stomach primarily include gastrointestinal stromal tumors (GISTs), leiomyomas, schwannomas, and lipomas, among which GISTs are the most common (3). Gastric leiomyoma and GISTs share similar imaging characteristics, often leading to clinical misdiagnosis and subsequent surgical intervention under the presumption of GISTs. While Gastric Leiomyoma typically presents in the fifth to seventh decades, cases in younger adults are extremely rare, this report describes a 28-year-old male, contrasting with the classical age-related epidemiological pattern.

## 2 Case report

A 28-year-old male underwent gastrointestinal endoscopy due to abdominal pain, which revealed a submucosal protrusion in the gastric fundus and chronic non-atrophic gastritis with erosion. Surgical intervention was recommended, but the patient declined. One month later, as the symptoms showed no improvement, the patient was admitted to the hospital for further treatment.

**Figure 1:**
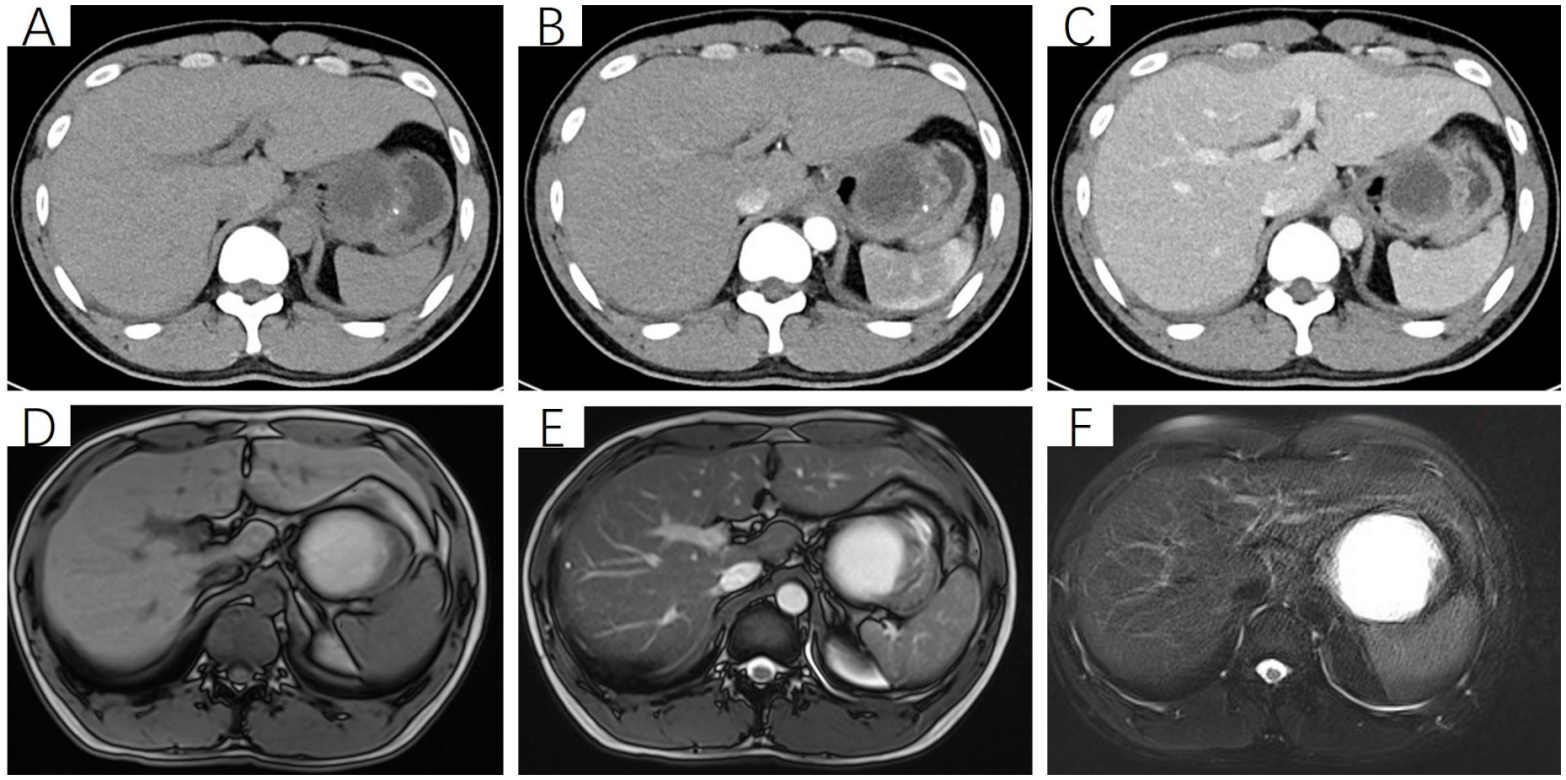
Non-contrast and contrast-enhanced CT scan of the upper abdomen. **(A)** Non-contrast scan reveals a cystic-solid mass beneath the left diaphragm, measuring approximately 63 mm × 64 mm × 58 mm, predominantly cystic with punctate calcifications in the solid component. The boundary between the lesion and the gastric wall of the fundus is indistinct; **(B, C)** Contrast-enhanced scan shows the cystic-solid mass, with significant enhancement of the solid component and no enhancement of the cystic component. **(D)** T1-weighted imaging (T1WI) reveals a round, cystic-solid mass in the gastric fundus, measuring approximately 63 mm × 64 mm × 58 mm. The cystic component appears hyperintense, while the solid component shows isointense signal. **(E, F)** T2-weighted imaging (T2WI) and T2-weighted fat-suppressed imaging (T2FS) demonstrate that the cystic component of the mass appears hyperintense, while the solid component remains isointense.

**Figure 2:**
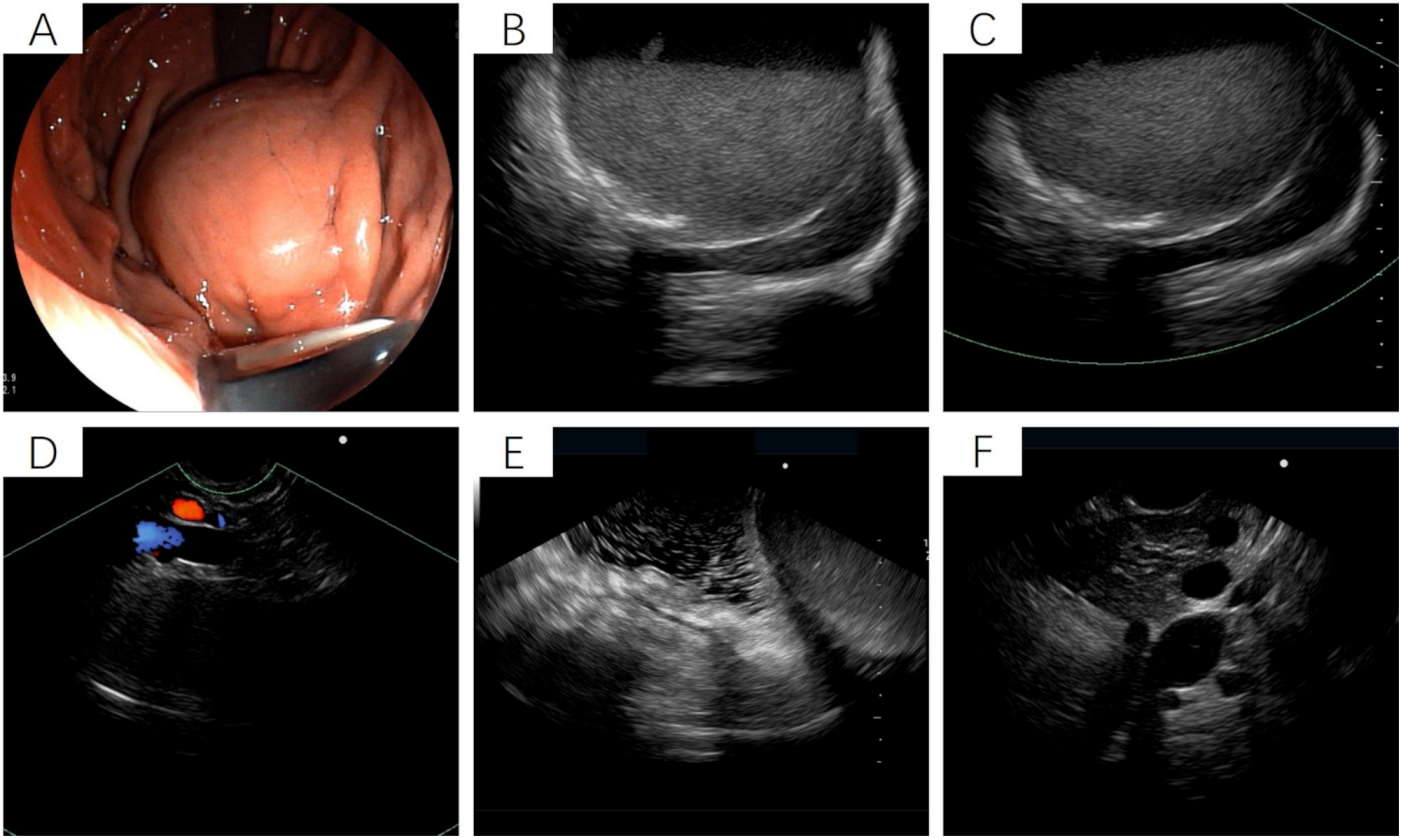
Endoscopic ultrasound of the stomach. **(A)** Gastroscopy reveals a raised lesion in the gastric fundus and body, presenting as a slope-like structure with no clear boundary from the surrounding tissue. Local mucosal edema is evident, showing nodular changes. No obvious varicose veins are observed in the fundus and body regions. **(B-F)** Ultrasound imaging shows a round, mixed-echo lesion in the splenic hilar region, with a cross-sectional size of approximately 73.8 mm × 56.7 mm. The lesion contains solid hyperechoic and liquid anechoic components, with a ratio of approximately 6:4. Color Doppler flow imaging (CDFI) reveals no significant blood flow signals in the cystic wall of the lesion.

**Figure 3:**
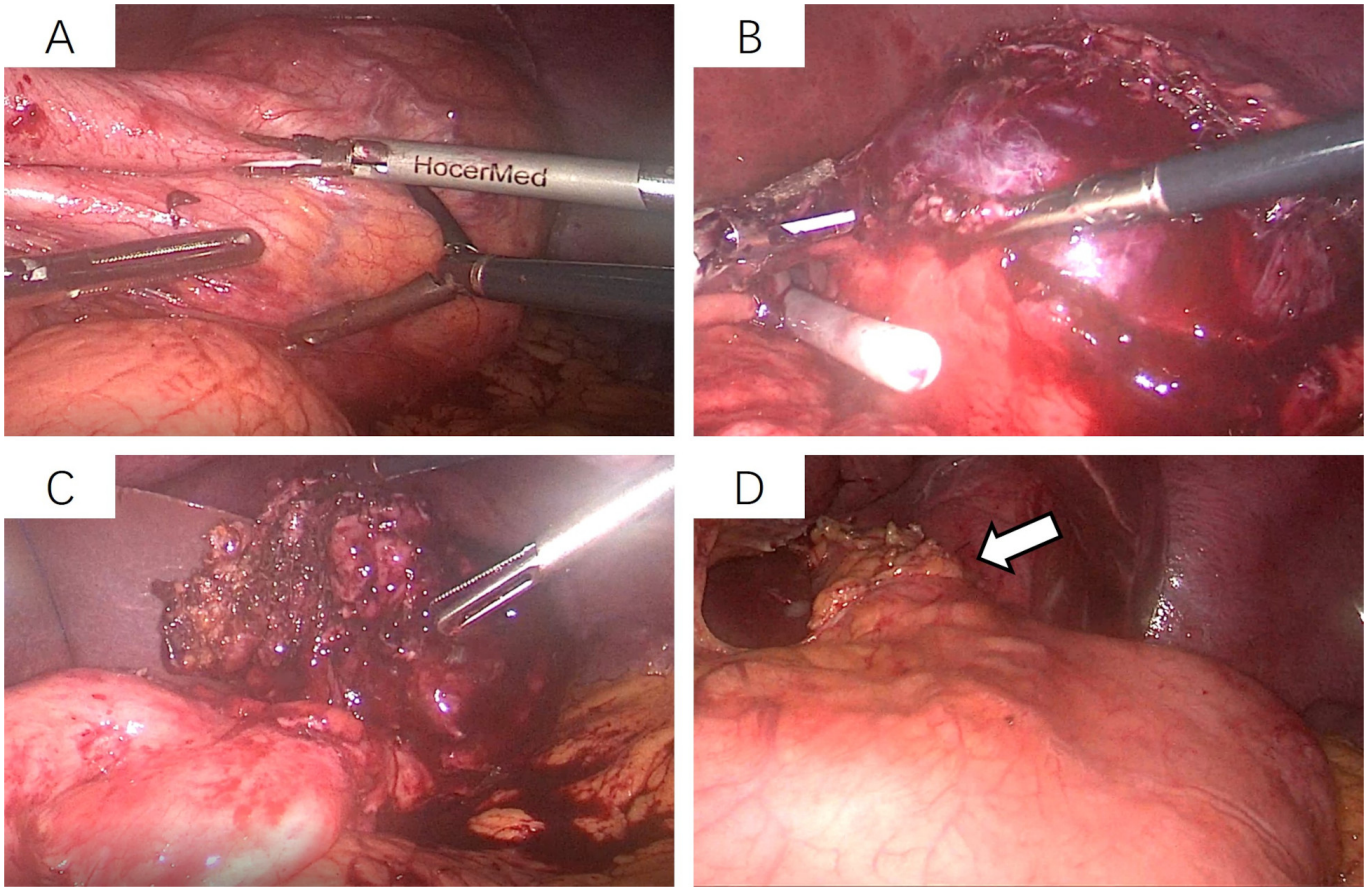
Laparoscopic resection of the gastric fundus tumor. **(A)** The tumor is located near the cardia of the gastric fundus, measuring approximately 6 × 7 cm, and is relatively mobile; **(B, C)** The gastric wall on the greater curvature side near the tumor is incised using an ultrasonic scalpel, and the tumor is completely excised along its margins. **(D)** After tumor removal, the edge of the gastric fundus is approximately 2 cm from the cardia, followed by continuous full-thickness suturing of the gastric wall.

**Figure 4:**
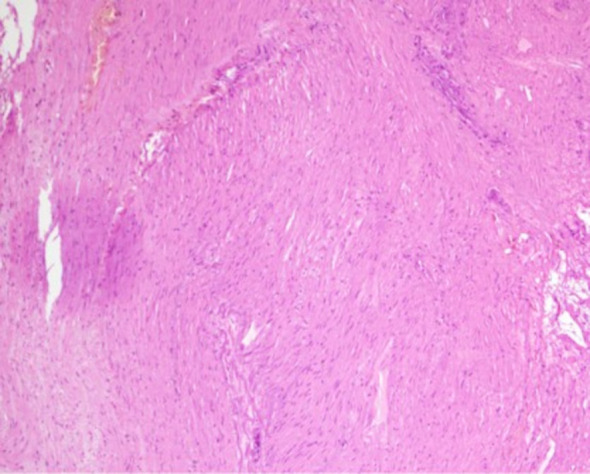
Postoperative pathological findings. (Gastric fundus mass) A thickened area is observed beneath the mucosa, with spindle cell proliferation in some regions. Focal necrosis and aggregation of foamy histiocytes are noted, accompanied by fibrosis and calcification. Immunohistochemical staining results: Vimentin (+), Actin (+), SMA (+), Desmin (+), Caldesmon (+), SDHB (+), S-100 (partial cells +), HMB45 (-), SOX-10 (-), GFAP (-), CD56 (-), Syn (-), CD117 (-), DOG-1 (-), CD34 (-), pan-CK (-), Ki-67 (+, 2%).

After admission, the patient underwent non-contrast and contrast-enhanced abdominal computed tomography (CT) scans, which revealed a cystic lesion measuring approximately 63mm × 64mm × 58mm below the left diaphragm. The lesion exhibited a thickened wall with curvilinear calcifications. On contrast-enhanced imaging, the wall of the lesion showed enhancement, and the boundary between the lesion and the gastric wall of the fundus was indistinct (**Figures 1A-C**). Endoscopic ultrasonography (EUS) revealed a raised lesion in the gastric fundus and body, presenting as a slope-like structure with no clear boundary from the surrounding tissue. Local mucosal edema was evident, showing nodular changes. On ultrasound, a round, mixed-echo lesion was detected in the splenic region, measuring approximately 73.8 × 56.7 mm in cross-section. The lesion contained both solid hyperechoic and liquid anechoic components, with a ratio of approximately 6:4. The pancreatic parenchyma exhibited slightly coarse echotexture with patchy areas (**Figure 2**). Magnetic resonance imaging (MRI) of the upper abdomen revealed a round-like mass with a diameter of approximately 6.3 cm in the gastric fundus. The mass exhibited short T1 and long T2 signal intensity, with a fluid-fluid level visible internally. Diffusion-weighted imaging showed restricted diffusion, and there was no significant enhancement on contrast-enhanced imaging (**Figures 1D-F**). Routine blood tests, coagulation function, tumor markers, and chest X-ray showed no abnormalities. The patient accepted laparoscopic resection of the gastric fundus tumor. Intraoperative exploration revealed no significant abnormalities in the liver, gallbladder, spleen, kidneys, colon, small intestine, greater omentum, abdominal wall, or pelvic cavity, and no suspicious nodules were observed in the abdominal cavity. The tumor was located near the cardia of the gastric fundus, measuring approximately 6 × 7 cm (**Figure 3A**), and was relatively mobile. A local resection of the gastric fundus tumor was performed: the gastric wall was incised using an ultrasonic scalpel near the greater curvature of the gastric fundus tumor, and the tumor was completely excised en bloc. After tumor resection, the margin of the gastric fundus was approximately 2 cm from the cardia (**Figures 3B, C**). The gastric wall was then continuously sutured in full thickness (**Figure 3D**). Intraoperative frozen section analysis suggested a “spindle cell tumor.” Postoperative pathological results confirmed the diagnosis of (gastric fundus tumor) leiomyoma. Immunohistochemical staining results showed Desmin (+) and SMA (+), while the expressions of proto-oncogenes CD117, CD34, and Dog-1 were negative (**Figure 4**). The patient recovered well and was discharged one week after surgery.

## 3 Discussion

The clinical manifestations of gastric tumors are diverse, encompassing symptoms related to local mass effects or infiltration (such as early satiety, nausea, vomiting, gastroesophageal reflux disease, hematemesis, or melena), systemic manifestations (such as anemia and weight loss), and pain caused by tumor invasion or metastasis (dull epigastric pain or radiating back pain) (4). Additionally, some patients may present with atypical or advanced features, including paraneoplastic syndromes (acanthosis nigricans, thrombophlebitis) or signs associated with metastatic lesions (enlarged left supraclavicular lymph nodes, ascites, hepatomegaly, jaundice) (5). Specific types of gastric tumors (such as gastric neuroendocrine tumors or gastrointestinal stromal tumors) may also be accompanied by characteristic syndromes (e.g., carcinoid syndrome) or acute complications (e.g., intra-abdominal hemorrhage) (6).

Gastric leiomyomas are relatively rare in clinical practice. They originate from smooth muscle cells of the gastric wall and exhibit slow growth, typically presenting as benign tumors (1). It is a benign tumor that grows beneath the mucosal layer, accounting for approximately 2.5% of gastric tumors (7). Gastric leiomyomas lack specific indicators in the early stages of onset and are easily confused with other stromal-derived tumors, which can lead to missed diagnoses and misdiagnoses (8). In terms of treatment, gastric leiomyomas differ significantly from other tumors in both therapeutic approaches and prognosis. Therefore, understanding the imaging and pathological characteristics of gastric leiomyomas, as well as the principles of treatment, is of great importance for improving diagnostic accuracy and avoiding inappropriate surgical interventions. Among these, gastric leiomyomas and GISTs are both common mesenchymal tumors of the stomach. Although their clinical manifestations are similar, their biological behaviors differ significantly, leading to distinct treatment plans and follow-up strategies. Gastric stromal tumors have malignant potential, with a high risk of recurrence and metastasis, and a poorer prognosis, whereas gastric leiomyomas are typically benign lesions. Therefore, it is crucial to emphasize the differential diagnosis between gastric leiomyomas and gastric stromal tumors in clinical practice (9).

Gastric leiomyomas frequently occur in the gastric cardia. Due to the anatomical constraints of the cardia, the longitudinal diameter of the tumor often aligns parallel to the gastric wall. As a result, some scholars recommend using a longitudinal diameter-to-transverse diameter ratio of >1.2 as one of the diagnostic criteria to differentiate GISTs (10). Studies have reported that gastric leiomyomas often present on imaging as solitary, well-defined, round nodules in the gastric fundus. On CT scans, they typically exhibit homogeneous density and demonstrate uniform enhancement after contrast administration. In contrast, GISTs often show heterogeneous density on non-contrast CT scans and significant enhancement after contrast administration. If the tumor is large and accompanied by necrosis, the enhancement pattern may appear heterogeneous (11). EUS can clearly visualize the layered structure of the gastrointestinal wall and has become one of the primary diagnostic tools for evaluating elevated lesions of the gastrointestinal mucosa. Under EUS, gastric leiomyomas are frequently found in the cardia and gastric fundus, typically presenting with more homogeneous echogenicity. If a submucosal tumor in the stomach is located in the cardia or gastric fundus and exhibits homogeneous echogenicity on EUS, gastric leiomyoma should be considered as the primary diagnosis (12). Some scholars suggest that, based on EUS imaging comparisons between gastric leiomyomas and GISTs, the presence of ulceration or surface depression on the tumor is more indicative of a gastric stromal tumor. On the other hand, if the tumor is located in the cardia, the likelihood of it being a gastric leiomyoma is twice as high as that of a gastric stromal tumor (13).

Gastric leiomyomas and gastrointestinal stromal tumors (GISTs) share overlapping imaging features on CT and MRI, complicating their differential diagnosis. FDG PET/CT aids in differentiating these two entities by evaluating glucose metabolism (14). Among 18F-FDG PET/CT metabolic parameters, the maximum standardized uptake value (SUVmax) represents the highest focal FDG uptake. Gastric leiomyomas, being benign, typically show low FDG uptake (SUVmax < 2.0), whereas GISTs, owing to their malignant potential, frequently exhibit higher uptake (SUVmax > 2.5). This metabolic disparity is particularly crucial when conventional imaging modalities (e.g., CT/MRI) yield indeterminate findings (15). In cases of necrosis or cystic degeneration, large gastric leiomyomas may exhibit CT/MRI features resembling GISTs (10). Here, FDG PET/CT becomes indispensable: while GISTs retain high FDG uptake in viable solid components despite necrosis, leiomyomas demonstrate metabolic inactivity in necrotic areas, with residual solid portions maintaining low SUVmax (16). In GIST management, FDG PET/CT also plays a pivotal role. In large or unresectable GISTs undergoing imatinib neoadjuvant therapy, baseline PET/CT provides precise quantification of metabolic tumor burden. Importantly, it detects early treatment-induced SUVmax reductions, reflecting metabolic changes. These functional changes typically precede morphological shrinkage on conventional imaging, enabling earlier assessment of treatment response (17).

In terms of treatment, if the gastric leiomyoma is located within the muscularis mucosae layer of the stomach, endoscopic submucosal dissection (ESD) is considered the preferred treatment option (18). However, most patients have difficulty diagnosing preoperatively and are often misdiagnosed with GISTs. Given the malignant potential of GISTs, ensuring negative resection margins is critical, which typically leads to the adoption of wedge resection of the gastric wall. Nevertheless, since gastric leiomyomas frequently occur in the cardia and often involve the esophagogastric junction, wedge resection can easily result in anastomotic stenosis or leakage. As a result, a significant proportion of patients are compelled to undergo proximal gastrectomy. If a preoperative diagnosis confirms gastric leiomyoma, the likelihood of performing a cardia-preserving simple tumor resection increases significantly (19).

In addition to mesenchymal tumors, gastric leiomyoma should also be distinguished from epithelial malignancies, particularly gastric adenocarcinoma. Although both may appear as submucosal lesions on endoscopy, key differentiating features exist: gastric adenocarcinoma often presents as an irregular, ulcerated, and elevated mass, whereas gastric leiomyoma typically exhibits a smooth mucosal covering (20). On contrast-enhanced CT, gastric adenocarcinoma usually demonstrates asymmetric gastric wall thickening with heterogeneous (or layered) enhancement and perigastric fat infiltration, features not observed in leiomyoma (21). Endoscopic ultrasound (EUS) is critical for differentiation, as gastric adenocarcinoma typically arises from the mucosal layer and disrupts the gastric wall architecture, whereas leiomyoma originates from the muscularis propria layer while maintaining mucosal integrity (22). On immunohistochemistry, the absence of epithelial markers (CK7, CK20) along with strong positivity for smooth muscle markers (Desmin, SMA) can reliably rule out gastric adenocarcinoma (23).

## 4 Conclusion

We believe that the comprehensive analysis of preoperative CT, MRI, and endoscopic ultrasonography can significantly improve the diagnostic accuracy of gastric leiomyoma by differentiating it from gastric stromal tumors and gastric adenocarcinoma. In this case, we performed a cardia-preserving resection of the gastric fundus tumor. After tumor removal, the edge of the gastric fundus was approximately 2 cm from the cardia, ensuring sufficient gastric wall for suturing and reducing the risk of postoperative stenosis. For mesenchymal tumors of the stomach, if preoperative diagnostic evidence strongly suggests gastric leiomyoma, adopting a cardia-preserving simple tumor resection is more conducive to patient recovery and postoperative quality of life.

## Data Availability

The original contributions presented in the study are included in the article/Supplementary Material. Further inquiries can be directed to the corresponding author.
